# Methcathinone and 3-Fluoromethcathinone Stimulate Spontaneous Horizontal Locomotor Activity in Mice and Elevate Extracellular Dopamine and Serotonin Levels in the Mouse Striatum

**DOI:** 10.1007/s12640-018-9973-4

**Published:** 2018-10-30

**Authors:** Jakub Wojcieszak, Dariusz Andrzejczak, Adam Wojtas, Krystyna Gołembiowska, Jolanta B. Zawilska

**Affiliations:** 1 Department of Pharmacodynamics, Medical University of Łódź, Muszyńskiego 1, 90-151 Łódź, Poland; 2 Department of Pharmacology, Institute of Pharmacology, Polish Academy of Sciences, Smętna 12, 31-343 Kraków, Poland

**Keywords:** Methcathinone, 3-Fluoromethcathinone, Spontaneous locomotor activity, Microdialysis, Dopamine, Serotonin, Novel psychoactive substances

## Abstract

Methcathinone (MC) and 3-fluoromethcathinone (3-FMC) are well-known members of the synthetic cathinone derivatives, the second most abused group of novel psychoactive substances (NPS). They are considered as methamphetamine-like cathinones, as they elicit their psychostimulatory effects via inhibition of monoamine uptake and enhanced release. The present study examines the effects of MC and 3-FMC on the spontaneous locomotor activity of mice and extracellular levels of dopamine and serotonin in the mouse striatum. Both MC and 3-FMC produced a dose-dependent increase of horizontal locomotor activity, but no significant changes in rearing behavior were observed. The locomotor stimulation induced by MC and 3-FMC is mediated by activation of dopaminergic neurotransmission, as selective D_1_-dopamine receptor antagonist, SCH 23390, abolished the effects of both drugs. In line with pharmacological data obtained by previous in vitro studies, MC and 3-FMC produced potent increases of extracellular dopamine and serotonin levels in the mouse striatum. Taken together, results presented within this study confirm previous findings and expand our knowledge on the pharmacology of MC and 3-FMC along with their behavioral effects.

## Introduction

Synthetic cathinones form an ever-growing group of novel psychoactive substances (NPS). Since their appearance on the clandestine market in the mid-2000s, novel substances belonging to this group have been introduced every year and a total number of 130 synthetic cathinones have been detected by the end of 2017. Notably, synthetic cathinones are among the most popular NPS, constituting 33% of the total seizures of NPS reported to the EU Early Warning System in 2016 (EMCDDA 2018). The group consists of diverse derivatives of the naturally occurring precursor, cathinone, which is a keto-analog of amphetamine and an active ingredient of *Catha edulis*, a plant traditionally chewed in the countries of Eastern Africa and the Arabian Peninsula (Patel 2018). Prominent synthetic cathinones include 4-methylmethcathinone (mephedrone), mexedrone, methylone, methcathinone (MC), 3-fluoromethcathinone (3-FMC), 4-chloromethcathinone (4-CMC), 4-chloroethcathinone (4-CEC), and brephedrone (4-BMC), as well as many analogs containing a pyrrolidine ring, such as 3,4-methylenedioxypyrovalerone (3,4-MDPV) and α-pyrrolidinopentiophenone (α-PVP) (Grifell et al. 2017; Liechti 2015; Zawilska and Wojcieszak 2013, 2017).

Generally, synthetic cathinones evoke their psychoactive effects by enhancing monoaminergic neurotransmission involving dopamine (DA), norepinephrine (NE), and serotonin (5-HT) via various mechanisms, including inhibition of monoamine reuptake, release of neurotransmitters stored inside synaptic vesicles and direct interaction with receptors, although the members of cathinones differ in their specificity for particular monoamines and mechanisms of interaction with their transporters (Simmler et al. 2013). It is well established that drugs acting on NE and DA rather than on 5-HT neurotransmission present greater sympathomimetic and reinforcing effects, while compounds enhancing 5-HT transmission produce MDMA-like empathogenic effects and disturbances in thermoregulation (Liechti 2015; Rickli et al. 2015; Simmler et al. 2013, 2014). Hence, the expected effects after administration of cathinones resemble these produced by amphetamine, methamphetamine and cocaine, or MDMA and include increased alertness and awareness, psychomotor agitation, loss of fatigue for pure psychostimulants, and increased sociability and empathy, along with intensification of sensory experiences and moderate sexual arousal for empathogens (Cozzi et al. 2013; Zawilska and Wojcieszak 2013, 2017). During their relatively short presence on the recreational drug market, synthetic cathinones were found to be the causal factor in numerous acute intoxications, some of which resulted in fatalities (Karila et al. 2015; Marinetti and Antonides 2013; Prosser and Nelson 2012; Zawilska and Wojcieszak 2013, 2017). Additionally, many synthetic cathinones, such as 3-FMC, 4-methylethcathinone (4-MEC), 3,4-MDPV, mephedrone, methylone, naphyrone, pentedrone, and α-PVP, together with α-PVP derivatives obtained by an alkyl side chain elongation and phenyl ring substitution, were found to be cytotoxic in vitro against neuronal and liver cells (den Hollander et al. 2014, 2015; Luethi et al. 2017; Matsunaga et al. 2017a, b; Siedlecka-Kroplewska et al. 2014, 2018; Valente et al. 2016a, b, 2017a, b; Wojcieszak et al. 2016, 2018a). Typical intoxication with synthetic cathinones produces symptoms associated with the cardiovascular (increased heart rate, arrhythmias, elevated blood pressure, and chest pain) and central nervous systems (dizziness, disorientation, insomnia, seizures, delusions, panic attacks, aggression, memory loss, anxiety, hallucinations), while overdose of compounds endowed with strong serotomimetic activity may result in a life-threatening serotonin syndrome and hyperthermia (Karila et al. 2015; Liechti 2015; Madras 2017; Prosser and Nelson 2012; Rickli et al. 2015; Zawilska and Wojcieszak 2013, 2017).

Previous works utilizing rodent models revealed substantial alternations of animals’ behavior and physiology caused by methcathinone and its substituted analogs (Aarde et al. 2015; Anneken et al. 2017, 2018; Bonano et al. 2014; Cozzi et al. 2013; Gatch et al. 2015a, b; Glennon et al. 1987; Kaizaki et al. 2014; Marusich et al. 2012, 2014; Meng et al. 1999; Shortall et al. 2013), which include the following:

- Psychomotor stimulation produced by both MC and its substituted analogs, including, among others, 3-FMC, shown as an increase of the spontaneous horizontal locomotor activity in mice and rats and increased incidence of stereotypies in mice. It is worth mentioning that members of α-pyrrolidinophenones, a distinct sub-group of synthetic cathinones characterized by the presence of pyrrolidine ring in place of amine group, such as 3,4-MDPV and α-PVP, produced a strong increase of locomotor activity in mice and rats. The potency of locomotor stimulation induced by α-pyrrolidinophenones was highly influenced by the length of alpha aliphatic side chain and mediated by D_1_-dopamine receptor stimulation.
- Signs of neurotoxicity and a potential for withdrawal effects as MC caused decrease of striatal DA, DAT, and tyrosine hydroxylase 48 h after treatment in mice. Additional neurotoxic effects have been reported for 3-FMC and include hypersalivation and decrease of motor coordination.
- Changes in thermoregulation as MC increased the rectal temperature in rats and increased the core body temperature in mice followed by the decrease at the highest tested dose (80 mg/kg).
- Addictive potential of MC and 3-FMC as they substituted for amphetamine, methamphetamine, and cocaine in rats trained to discriminate these substances from saline, while MC additionally produced dose- and time-dependent facilitation of intracranial self-stimulation in rats.

**Fig. 1:**
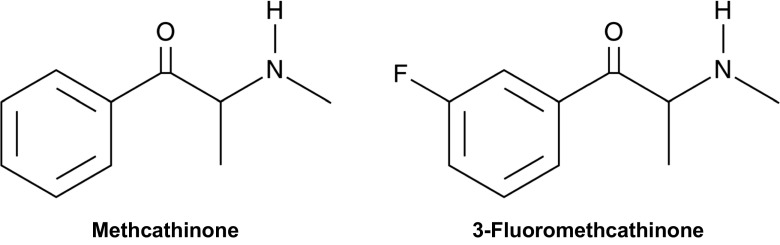
Structures of methcathinone and 3-fluoromethcathinone

In vitro studies show that both MC and 3-FMC are potent DA and NE uptake inhibitors with negligible effects on 5-HT uptake. Both drugs are endowed with the ability to release DA and NE. MC is also able to release 5-HT from pre-loaded cells, albeit with low potency. These activities of MC and 3-FMC support their classification as methamphetamine-like cathinones (Cozzi et al. 2013; Liechti 2015; Simmler et al. 2013, 2014). However, the results obtained from microdialysis studies of monoamine concentrations in the rat nucleus accumbens do not unequivocally indicate whether MC is capable of increasing extracellular 5-HT levels in vivo (Cozzi et al. 2013) or not (Suyama et al. 2016). Therefore, the aim of the present study was to obtain a broader view of in vivo pharmacologic profile of methcathinone and its common derivative, 3-fluoromethcathinone (Fig. 1), by assessing their effects on extracellular DA and 5-HT levels in the striatum and spontaneous locomotor activity in mice as an indicator of psychostimulant action.

## Materials and Methods

### Drugs and Reagents

Methcathinone [MC, 2-(methylamino)-1-phenyl-1-propanone] and 3-fluoromethcathinone [3-FMC, 2-(methylamino)-1-(3-fluorophenyl)-1-propanone] were purchased in the form of hydrochloride salts from Cayman Chemical (Ann Arbor, MI, USA). Isotonic solution of saline (0.9% NaCl) for injections was purchased from Polska Grupa Farmaceutyczna (Łódź, Poland). Selective D_1_-dopamine receptor antagonist, SCH 23390 (SCH, 7-chloro-8-hydroxy-3-methyl-1-phenyl-2,3,4,5-tetrahydro-1*H*-3-benzazepine), in the form of hydrochloride salt, was purchased from Sigma Aldrich (Poznań, Poland). All chemicals used for high-performance liquid chromatography (HPLC) were purchased from Merck (Warsaw, Poland). Ketamine hydrochloride and xylazine used for anesthesia were purchased from Biowet (Puławy, Poland).

### Animals

All housing conditions and experimental procedures were in accordance with the European Union guidelines regarding the care and use of laboratory animals (European Communities Council Directive of September 2010 (2010/63/EU)).

For all experiments, male C57BL/6J mice at approx. 9–12 weeks of age were used. Animals were housed in a sound-attenuated chamber, four per cage, with automatic 12-h light/dark cycles (lights on at 06:00 a.m.), with free access to drinking water and standard food pellets. All experiments were performed during the light cycle (08:00–14:00). In all experiments, each mouse was used only once, obtained one injection of either MC or 3-FMC and was drug-naïve prior to the treatment.

### Locomotor Activity

Experiments were conducted as previously described (Wojcieszak et al. 2018b). Briefly, tested compounds were dissolved in 0.9% saline and injected subcutaneously (s.c.) in a volume of 0.1 mL/10 g of body mass. Each group consisted of eight randomly assigned mice. The assessment of the influence of drugs on locomotor activity was conducted using the following groups: vehicle (0.9% saline), MC (1, 3 and 10 mg/kg), and 3-FMC (1, 3, 10 mg/kg). For this part of the study, one vehicle group consisting of eight mice was used as a control for both MC and 3-FMC in all doses in order to minimalize total number of experimental animals. Additionally, the impact of the D_1_-dopamine receptors on the drug-induced locomotor stimulation was investigated by pretreatment of animals with SCH 23390 (SCH; 0.06 mg/kg), a selective D_1_-DA receptor antagonist, 30 min before MC or 3-FMC treatment. This set of experiments consisted of the following groups: vehicle + vehicle, SCH (0.06 mg/kg) + vehicle, vehicle + MC (10 mg/kg), SCH (0.06 mg/kg) + MC (10 mg/kg), vehicle + 3-FMC (10 mg/kg), and SCH (0.06 mg/kg) + 3-FMC (10 mg/kg). Vehicle + vehicle and SCH + vehicle groups were tested once and used for analysis of both MC and 3-FMC. Immediately after injection of the drug, the mice were placed for 120 min in the open-field locomotor activity measuring chambers (20.3 × 20.3 × 20.3 cm) included in the Opto-Varimex Auto-Track hardware (model 0271-002M, 143 Columbus Instruments, Columbus, OH, USA). Each chamber was equipped with sets of 16 infrared beams spaced by 1.3 cm, coupled with corresponding photodetectors located on the horizontal X and Y axes. A second set of identical infrared beams and photodetectors was placed on a higher layer in order to detect vertical movement of mice (rearing behavior). Experimental analysis was based on the counts of beam breaks on the bottom and top layers within 10-min intervals. Experiments were performed in a sound-attenuated room with a dim red light (invisible for rodents) from above.

### Brain Microdialysis

Experiments were conducted as previously reported (Wojcieszak et al. 2018b).

### Surgery and Microdialysis Procedure

Each group consisted of six randomly assigned mice. The animals were anesthetized with ketamine (7.5 mg/kg) and xylazine (1 mg/kg) and vertical microdialysis probes with a 2-mm-long active site (MAB 10.8.2.Cu; AgnTho’s, Lidingö, Sweden) were implanted into the striatum using the following coordinates: AP + 1.0, L + 1.8, V − 3.8 (Paxinos and Franklin 2008). On the following day, probe inlets were connected to a syringe pump (BAS, West Lafayette, IN, USA) which delivered an artificial cerebrospinal fluid composed of [mM]: NaCl 147, KCl 2.7, MgCl_2_ 1.0, CaCl_2_ 1.2; pH 7.4, at a flow rate of 1.5 μL/min. After 1 hour of washout, three basal dialysate samples were collected every 20 min. The animals were then injected s.c. with vehicle (0.9% saline), MC (3 and 10 mg/kg), or 3-FMC (3 and 10 mg/kg), and fraction collections continued for 180 min. One vehicle group was used for analysis of both MC and 3-FMC. At the end of the experiment, the mice were sacrificed, and their brains were isolated to confirm the location of the probes by histological examination.

### Analytical Procedure of Samples

DA and 5-HT contents in the dialysate fractions were analyzed by HPLC with electrochemical detection. Chromatography was performed using an Ultimate 3000 System (Dionex, Sunnyvale, CA, USA), a Coulochem III electrochemical detector (model 5300; ESA, Chelmsford, MA, USA) with 5020 guard cell, 5014B microdialysis cell, and Hypersil Gold-C18 analytical column (3 × 100 mm; Thermo Scientific, Waltham, MA, USA). The mobile phase was composed of 0.1 M potassium phosphate buffer adjusted to pH 3.6, 0.5 mM EDTA, 16 mg/L 1-octanesulfonic acid sodium salt, and 2% methanol. The flow rate during analysis was set at 0.7 mL/min. The applied potential was + 600 mV for the guard cell, and E1 = − 50 mV and E2 = + 300 mV for the microdialysis cells, with a sensitivity set at 50 nA/V. The chromatographic data was processed by Chromeleon v. 6.80 (Dionex) software, run on a personal computer.

### Data Analysis

#### Locomotor Activity

Statistical analysis was performed using GraphPad Prism 6.0 software (GraphPad, San Diego, CA, USA). Locomotor activity was expressed as the total distance traveled (cm) and total number of rearings during each 10-min bins during the 120-min experiments. Statistical significance was determined using a two-way repeated measures analysis of variance (treatment; time after injection) followed by Tukey’s post hoc test. Additionally, one-way ANOVA followed by Tukey’s or Sidak’s post hoc tests was performed to evaluate total distance (cm) and total count of vertical beam breaks during each 120-min session. The results were recognized as statistically significant when *P* < 0.05.

#### Microdialysis

Statistical analysis was performed using STATISTICA V12.0 software (StatSoft, Kraków, Poland). Levels of DA and 5-HT are expressed as percentage of the basal level, fixed to 100%. The statistical significance was calculated using a repeated measures ANOVA over 20-min bins for the time course, followed by Tukey’s post hoc test. To analyze differences in AUC, one-way ANOVA was performed, followed by Tukey’s post hoc test. Results were recognized as statistically significant when *P* < 0.05.

## Results

### Effects of MC and 3-FMC on the Spontaneous Locomotor Activity of Mice

**Fig. 2:**
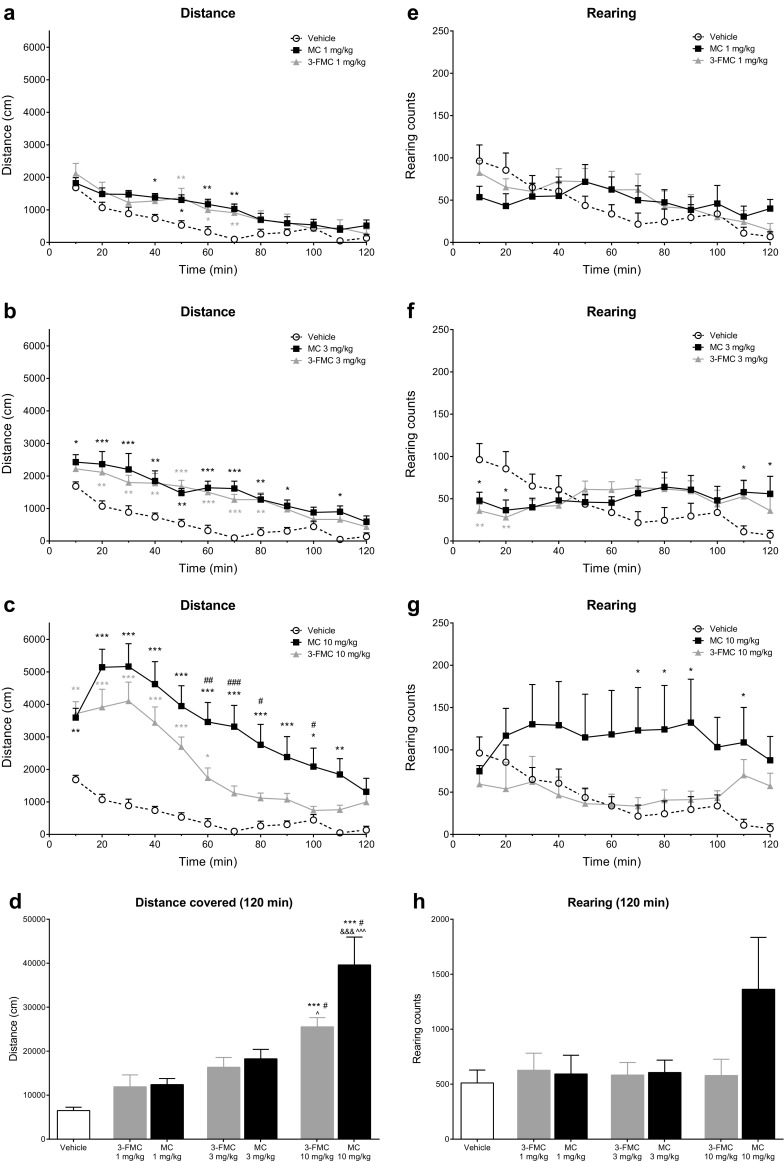
Effects of methcathinone (MC; 1, 3, 10 mg/kg) and 3-fluoromethcathinone (3-FMC; 1, 3, 10 mg/kg) on the spontaneous horizontal (panels **a**, **b**, **c**) and vertical (panels **e**, **f**, **g**) activity of mice measured in 10-min bins, and on total horizontal (panel **d**) and vertical (panel **h**) locomotor activity during 120 min. Data presented as mean ± SEM (*n* = 8). ***P < 0.001; **P < 0.01; *P < 0.05 vs. vehicle; ^###^*P* < 0.001; ^##^*P* < 0.01; *P* < 0.05 MC vs. 3-FMC; ^^^^^*P* < 0.001; ^^^*P* < 0.05 vs. 1 mg/kg; ^&&&^*P* < 0.001 vs. 3 mg/kg, Tukey’s post hoc test. For all analyses, the same control group was used; therefore, its data reappears on panels **a**–**c** and **e**–**g**

Both methcathinone (MC) and 3-fluoromethcathinone (3-FMC) administration resulted in a dose-dependent increase of horizontal spontaneous activity (*F*_3,28_ = 17.71; *P* < 0.0001 for MC) and (*F*_3,28_ = 15.12; *P* < 0.0001 for 3-FMC) (Fig. 2).

Treatment of mice with a 1 mg/kg dose of MC and 3-FMC of 1 mg/kg did not significantly affect their horizontal and vertical activity (*F*_2,21_ = 3.293; *P* = 0.0570) and (*F*_2,21_ = 0.1585; *P* = 0.8545), respectively (Fig. 2a, e). Both horizontal and vertical activities were significantly affected by time (*F*_11,23_ = 44.75; *P* < 0.0001); (*F*_11,231_ = 12.13; *P* < 0.0001) and interaction between time and treatment (*F*_22,231_ = 1.745; *P* = 0.0236 and *F*_22,231_ = 2.688; *P* = 0.0001), respectively. Neither MC nor 3-FMC caused a significant change of the total distance traveled and total rearing counts during the 120 min of the experiment (Fig. 2d, h). Within the 10-min time bins, MC significantly elevated horizontal activity between 40 and 70 min post injection, while 3-FMC did so between 50 and 70 min post injection. Neither compound had any effect on the rearing activity within 10-min time bins. No significant differences between MC and 3-FMC were observed regarding the horizontal and vertical activities of mice, neither for the 10-min bins nor the 120-min totals.

Treatment of mice with 3 mg/kg MC and 3-FMC significantly influenced horizontal (*F*_2,21_ = 11.79; *P* = 0.0004), but not vertical locomotor activity (*F*_2,21_ = 0.1860; *P* = 0.8316) (Fig. 2b, f). Moreover, horizontal activity was also significantly affected by time (*F*_11,231_ = 33.91; *P* < 0.0001) and interaction between factors (*F*_22,231_ = 1.733; *P* = 0.0250), while rearing behavior was affected only by interaction (*F*_22,231_ = 5.014; *P* < 0.0001), and not by time (*F*_11,231_ = 1.465; *P* = 0.1456). Within 10-min bins, MC caused a significant increase of horizontal locomotor activity in 0–90 and 100–110 min post injection intervals, while locomotor stimulation caused by 3-FMC started at 10 min post injection and persisted until 80 min post injection. It is noteworthy that both compounds used at 3 mg/kg caused a significant elevation of horizontal locomotor activity that persisted for a longer duration than in the case of 1 mg/kg dose. Rearing behavior was significantly increased only during 100–120 min after treatment with 3 mg/kg MC, while both MC and 3-FMC at 3 mg/kg markedly decreased rearing behavior during the first 20 min post injection. Neither compound at 3 mg/kg significantly altered the total distance traveled or total rearing counts during 120 min of the experiment (Fig. 2d, h). Also, no significant differences in horizontal and vertical activities between groups receiving 3 mg/kg MC and 3-FMC were found in the analysis of 10-min bins or total scores after 120-min observation.

Injection of MC and 3-FMC at a dose of 10 mg/kg led to the potent stimulation of horizontal locomotor activity (Fig. 2c), which was significantly influenced by treatment (*F*_2,21_ = 18.46; *P* < 0.0001), time (*F*_11,231_ = 47.52; *P* < 0.0001), and interaction between factors (*F*_22,231_ = 6.593; *P* < 0.0001). On the other hand, vertical activity was not affected by either treatment (*F*_2,21_ = 2.601; *P* = 0.0979) or time (*F*_11,231_ = 1.557; *P* = 0.1128); however, a significant effect of interaction between factors (*F*_22,231_ = 1.913; *P* = 0.0100) was observed. Within 10-min bins, MC and 3-FMC significantly increased horizontal locomotor activity for 110 and 60 min, respectively, starting immediately after injection. Moreover, horizontal locomotor activity was significantly higher in the MC group vs. 3-FMC group in the 50–80 and 90–100 min post injection intervals. Rearing behavior was significantly increased only in the MC group in the 60–90 and 100–110 min post injection intervals (Fig. 2g). Both compounds at 10 mg/kg significantly elevated the total distance traveled through the 120 min of the experiment comparing to the vehicle and 1 mg/kg groups (Fig. 2d). Additionally, the distance traveled after treatment with 10 mg/kg MC was also significantly higher than that in the 3 mg/kg MC group and the 10 mg/kg 3-FMC group. No significant differences in total rearing counts during 120 min were found after treatment with MC or 3-FMC at 10 mg/kg (Fig. 2h).

### Role of D_1_-Dopamine Receptors in MC- and 3-FMC-Induced Psychomotor Stimulation in Mice

**Fig. 3:**
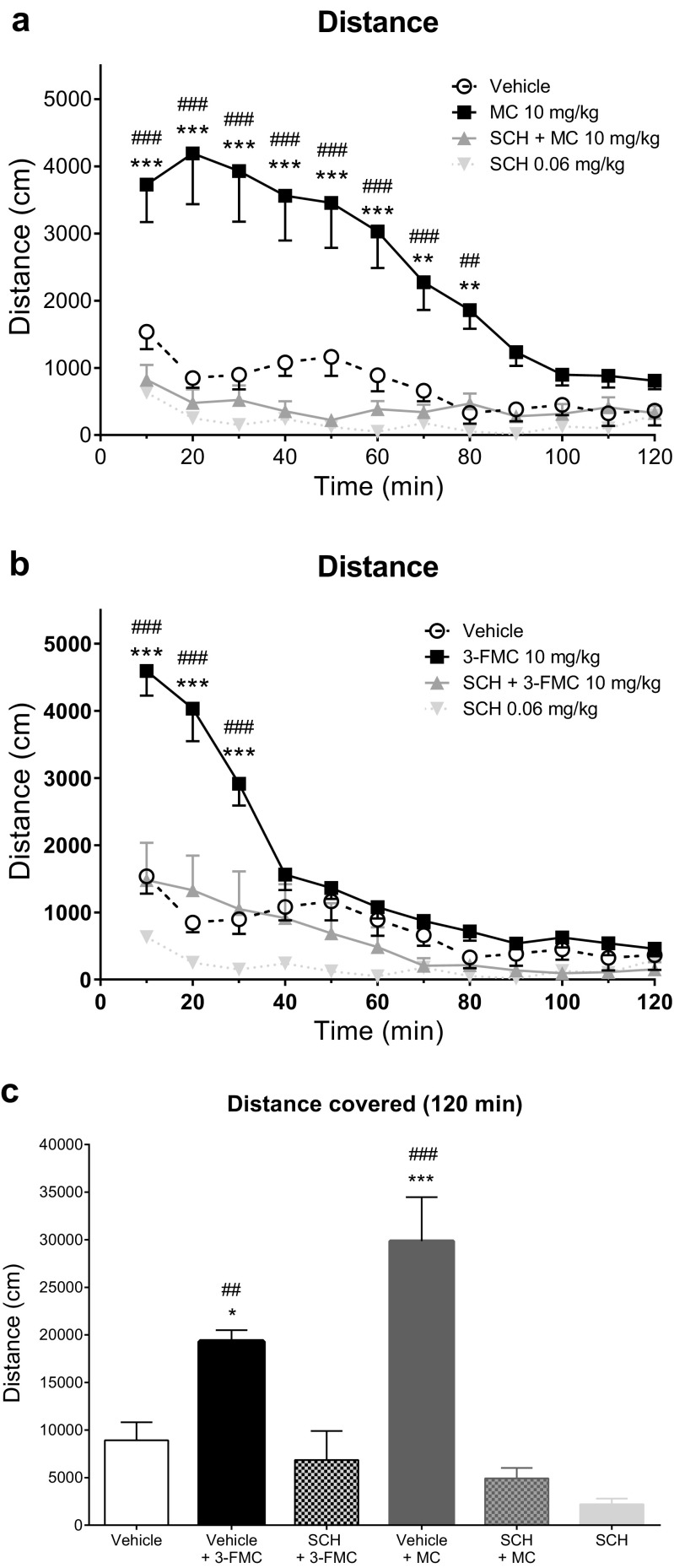
Effects of the SCH 23390 (SCH; 0.06 mg/kg) pretreatment followed by treatment with methcathinone (MC; 10 mg/kg) or 3-fluoromethcathinone (3-FMC; 10 mg/kg) on the horizontal activity of mice measured in 10-min bins (panels **a** and **b**, respectively), and on total horizontal activity during 120 min (panel **c**). Data presented as mean ± SEM (*n* = 8). ***P < 0.001; **P < 0.01 vs. vehicle; ^###^*P* < 0.001; ^##^*P* < 0.01; ^#^*P* < 0.05 vs. SCH + MC or SCH + 3-FMC group, Tukey’s (panels **a** and **b**) or Sidak’s (panel **c**) post hoc test. For both compounds, one analysis containing single vehicle and single SCH group was performed. Separation of data into panels **a** and **b** was done in order to improve visual appearance; therefore, the same data reappears on panels **a** and **b**

Treatment of mice with a selective D_1_-dopamine receptor antagonist, SCH 23390 (0.06 mg/kg), 30 min prior to the administration of MC or 3-FMC led to the significant suppression of cathinone-induced horizontal locomotor activity (Fig. 3).

The analysis of groups receiving vehicle + vehicle, vehicle + MC (10 mg/kg), SCH (0.06 mg/kg) + MC (10 mg/kg), vehicle + 3-FMC (10 mg/kg), SCH (0.06 mg/kg) + 3-FMC (10 mg/kg), and SCH (0.06 mg/kg) + vehicle demonstrated that treatment (*F*_5,42_ = 17.87; *P* < 0,0001), time (*F*_11,462_ = 49.38; *P* < 0,0001), and interaction between factors (*F*_55,462_ = 9.051; *P* < 0,0001) had significant effects on the spontaneous horizontal locomotor activity of mice. MC- and 3-FMC-induced locomotor stimulation in mice was significantly blocked by pretreatment with SCH 23390 through the entire duration of both compounds action: 80 min for MC and 30 min for 3-FMC (Fig. 3a, b). No statistically significant difference between vehicle + vehicle and SCH + vehicle groups was observed at any time point.

Additionally, the total distance traveled for 120 min was significantly lower in the SCH + MC group than in the vehicle + MC group and in the SCH + 3-FMC group compared to vehicle + 3-FMC group. No difference was observed in the vehicle + vehicle vs. SCH + MC or vehicle + vehicle vs. SCH + 3-FMC groups, meaning that locomotor stimulation induced by MC and 3-FMC was totally abolished by the SCH pretreatment. No statistically significant difference was found between vehicle + vehicle and SCH + vehicle groups, indicating that decrease of locomotor activities observed in groups pretreated with SCH did not result solely from SCH’s catatonic effect (Fig. 3c).

### Effects of MC and 3-FMC on Extracellular DA and 5-HT Levels in the Mouse Striatum

Basal levels of extracellular monoamines (in pg/10 μL; mean ± SEM) were 6.5 ± 0.25 for DA (*n* = 30) and 0.7 ± 0.07 for 5-HT (*n* = 30).

**Fig. 4:**
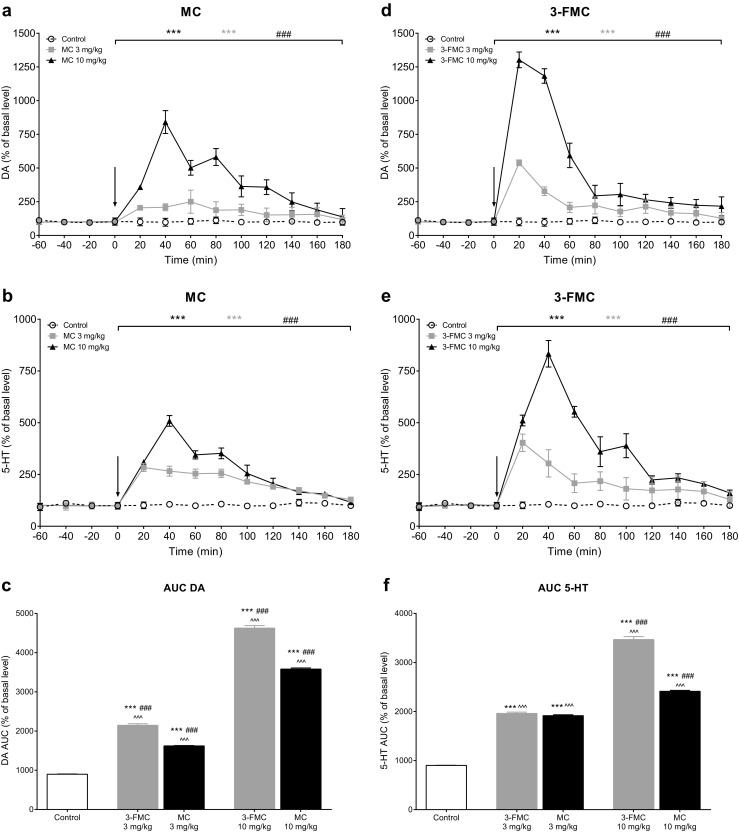
Effects of methcathinone (MC; 3, 10 mg/kg) and 3-fluoromethcathinone (3-FMC; 3, 10 mg/kg) on the extracellular DA and 5-HT levels in the mouse striatum, shown as a time course (panels **a**, **b**, **d**, **e**) or an area under the curve, AUC (panels **c**, **f**). Drug administration is indicated with an arrow. Data presented as mean ± SEM (*n* = 6). ***P < 0.001 vs. control group; ^###^*P* < 0.001 3 mg/kg vs. 10 mg/kg; ^^^^^*P* < 0.001 MC vs. 3-FMC, Tukey’s post hoc test. For both drugs and doses, one control group was used; therefore, its data reappears on panels **a** and **d**, and **b** and **e**

Both MC and 3-FMC caused significant increases of extracellular DA and 5-HT levels in the mouse striatum (Fig. 4).

Treatment with MC at doses of 3 and 10 mg/kg caused a significant elevation of extracellular DA levels in the mouse striatum (Fig. 4a, c). Maximal effects of ca. 250% and ca. 840% of the basal level were reached after 60 and 40 min post injection, respectively (Fig. 4a). Statistical analysis revealed that treatment (*F*_2,15_ = 5974.89; *P* < 0.0001), time (*F*_8,120_ = 349.52; *P* < 0.0001), and interaction between factors (*F*_16,120_ = 250.18; *P* = 0.0001) significantly affected extracellular DA levels. Extracellular levels of 5-HT in the mouse striatum were also significantly increased after treatment with both doses of MC, reaching maximal effects of ca. 280% of the basal level 20 min after treatment with 3 mg/kg MC and ca. 510% of the basal level 40 min after treatment with 10 mg/kg (Fig. 4b, f). Statistical analysis indicated that treatment (*F*_2,15_ = 2589.47; *P* < 0.0001), time (*F*_8,120_ = 182.02; *P* < 0.0001), and interaction between factors (*F*_16,120_ = 74.24; *P* < 0.0001) significantly affected extracellular 5-HT levels in the mouse striatum.

Extracellular levels of both DA and 5-HT were significantly elevated compared to the control group at each 20-min time point after treatment with either 3 mg/kg or 10 mg/kg MC (Fig. 4a, b).

Treatment with 3-FMC at 3 and 10 mg/kg also significantly elevated extracellular DA levels in the mouse striatum (Fig. 4d, f). Maximal effects of ca. 540% and ca. 1300% of the basal level were reached 20 min after injection with 3 mg/kg and 10 mg/kg 3-FMC, respectively. Repeated measures ANOVA indicated that treatment (*F*_2,15_ = 1931.81; *P* < 0.0001), time (*F*_8,120_ = 394.77; *P* < 0.0001), and interaction between factors (*F*_16,120_ = 200.76; *P* < 0.0001) had significant effects on extracellular DA levels.

Both doses of 3-FMC significantly increased extracellular 5-HT levels in the mouse striatum, with maximal effects of ca. 400% of the basal level 20 min after treatment with 3 mg/kg 3-FMC and ca. 830% of the basal level 40 min after treatment with 10 mg/kg 3-FMC (Fig. 4e). Repeated measures ANOVA revealed a significant impact of treatment (*F*_2,15_ = 1155.902; *P* < 0.0001), time (*F*_8,120_ = 131.057; *P* < 0.0001), and interaction between factors (*F*_16,120_ = 67.900; *P* < 0.0001) on the extracellular 5-HT levels in the mouse striatum.

Extracellular levels of both DA and 5-HT were significantly elevated comparing to the control group at each 20-min time point after treatment with either 3 mg/kg or 10 mg/kg 3-FMC (Fig. 4d, e).

Additional one-way ANOVA followed by Tukey’s post hoc test of the total effects measured as an area under the curve (AUC) for both monoamines revealed that MC and 3-FMC administered at doses of 3 mg/kg and 10 mg/kg significantly elevated extracellular levels of DA and 5-HT over the 180-min measurement (*P* < 0.001) (Fig. 4c, f). In all cases, the effects induced by doses of 10 mg/kg were significantly more pronounced than those induced by 3 mg/kg (*P* < 0.001). 3-FMC compared to MC produced significantly greater DA level elevation after either 3 mg/kg or 10 mg/kg, and significantly greater increases of 5-HT levels at 10 mg/kg (*P* < 0.001).

## Discussion

The present study confirms previous findings that both MC and 3-FMC produce dose-dependent increases of spontaneous horizontal locomotor activity in mice (Gatch et al. 2015a; Marusich et al. 2012). Enhancement of spontaneous locomotor activity in mice observed after treatment with MC and 3-FMC is less potent compared to effects of α-pyrrolidinophenone analogs, such as 3,4-MDPV and α-PVP. Both α-pyrrolidinophenones significantly increased the total distance covered during experiment, even at the dose of 1 mg/kg or 3 mg/kg, while MC and 3-FMC failed to do so at 1 and 3 mg/kg (Giannotti et al. 2017; Wojcieszak et al. 2018b). Additionally, in various studies, effects of 3,4-MDPV and α-PVP at 10 mg/kg persisted for 120–240 min (Gatch et al. 2013, 2015; Giannotti et al. 2017, Wojcieszak et al. 2018b), while in the current study effects of MC and 3-FMC at the same dose worn out after 60 and 110 min, respectively. These observations are in line with the study by Marusich et al. (2012), in which the effects of 3-FMC seem to be weaker compared to 3,4-MDPV and tend to terminate before 90 min at doses below 30 mg/kg.

Interestingly, neither MC nor 3-FMC significantly affected rearing behavior, an activity which was found to be intensively enhanced by three α-pyrrolidinophenone analogs, i.e., α-PVP, PV8, and PV9; members of a distinct branch of synthetic cathinones (Wojcieszak et al. 2018b). Moreover, a similar pattern of the dose-dependent increase of horizontal locomotor activity with no effect on vertical activity in mice was previously observed for methamphetamine (Wojcieszak et al. 2018b). In rodents, rearing is generally considered as an exploratory and hyperactive behavior. However, it is also used as a marker related to anxiety, with decreased rearing counts defined as a sign of elevated anxiety, as in this posture rodents are more vulnerable to predators (Ennaceur 2014; Eudave et al. 2018; Hoxha et al. 2019; Rodgers et al. 1997). Therefore, we assume that increased vertical locomotor activity after treatment with α-PVP, PV8, or PV9 resulted from an intense hyperactivity with concomitant suppression of anxiety, while effects of MC and 3-FMC can be described as a moderate locomotor stimulation, causing only a slight decline in the manifestation of anxiety-related behavior at the highest tested dose of MC (10 mg/kg). However, as we did not perform any specific tests for anxiety, aforementioned assumptions have to be taken as a hypothesis.

Our present findings support the view that increases in locomotor activity result from dopaminergic stimulation, as both drugs elevated extracellular DA levels in the mouse striatum in time periods corresponding to the observed locomotor stimulation, and the psychostimulatory effects of MC and 3-FMC were abolished by pretreatment of mice with the selective D_1_-DA receptor antagonist, SCH 23390.

To our knowledge, this is the first study comparing effects of 3-FMC with those evoked by MC. The choice of investigated compounds resembles the NPS-abuse pattern, as 3-FMC is commonly found in “research chemicals” compounds (Archer 2009; Odoardi et al. 2016), while MC is home-synthesized using OTC medications containing pseudoephedrine and potassium permanganate by drug abusers (de Bie et al. 2007; Iqbal et al. 2012; Sikk and Taba 2015). MC at 10 mg/kg produced significantly more pronounced locomotor stimulation than 10 mg/kg 3-FMC in terms of effect duration and distance traveled in various 10-min time bins, as well as during the whole observation. This observation may be seen as contrasting with the significantly higher AUC values for the extracellular DA levels in the mouse striatum obtained after injection with 3-FMC compared to MC. However, it should be noted that high AUC for DA levels reached after 3-FMC treatment is strongly influenced by a very high initial increase (ca. 1300% of the basal level), followed by a rapid decrease of DA levels. On the other hand, the elevation of DA levels in the mouse striatum induced by MC is more temperate—it reaches a lower maximum, but is maintained above 300% of the basal level for a longer period of time, thus causing locomotor stimulation of a longer duration. Additionally, lower locomotor stimulation induced by 3-FMC may be also attributed to the significantly more pronounced increase of 5-HT levels after 3-FMC treatment, combined with a similar duration, as DA over 5-HT selectivity is considered to be the major factor determining the psychostimulant/empathogen drug profile (Liechti 2015; Rickli et al. 2015; Simmler et al. 2013).

Both MC and 3-FMC are classified as methamphetamine-like cathinones, as they are potent DA and NE uptake inhibitors, with negligible effects on 5-HT uptake; however, both compounds produce a significant release of aforementioned catecholamines from pre-loaded cells (Liechti 2015; Cozzi et al. 2013; Simmler et al. 2013, 2014). These findings are supported by results obtained in our microdialysis studies in which MC and 3-FMC were found to potently increase extracellular levels of DA. While we are not aware of any study on the effects of 3-FMC on extracellular levels of monoamines in vivo, our results support previous findings of Cozzi et al. (2013), who found that MC significantly increases extracellular levels of DA and 5-HT in the rat nucleus accumbens. However, this contrasts with other findings indicating that MC elevated DA but not 5-HT extracellular levels in the rat nucleus accumbens (Suyama et al. 2016). It is worth noting that in the present study, MC was used in significantly higher doses in mice (3 and 10 mg/kg) than the 0.32 and 1 mg/kg used in rats (Suyama et al. 2016). Additionally, the authors of the previous study also found that 4-fluoromethcathinone (4-FMC), a positional isomer of 3-FMC, and other *para*-substituted methcathinone derivatives, elevate both DA and 5-HT levels in the rat nucleus accumbens (Suyama et al. 2016). Interestingly, as opposed to relatively low potency to release 5-HT in vitro (Cozzi et al. 2013), MC and 3-FMC induce considerable increase of extracellular levels of 5-HT. A similar phenomenon was reported before; compounds considered to be highly selective for DAT over SERT in vitro, such as methamphetamine and α-PVP along with its derivatives, were found to significantly increase extracellular 5-HT levels in rodent brains in vivo (Ago et al. 2011; Baumann et al. 2012; Matsumoto et al. 2014; Wojcieszak et al. 2018b). The possible explanation of this phenomenon may be the presence of a functional DA—5-HT crosstalk, meaning that both transporters reciprocally have a substantial ability to uptake the other monoamine. Furthermore, increased levels of extracellular DA may favor the efflux of intracellular 5-HT via SERT during the uptake of DA by DAT (Larsen et al. 2011).

It should be emphasized that 3-FMC, despite causing relatively weaker locomotor stimulation compared to α-PVP, as discussed above, when used at the 10 mg/kg dose induced the increase of extracellular DA levels in mouse striatum with a higher maximal effect than 10 mg/kg α-PVP (ca. 1300% vs. ca. 630%), but with a sooner decline (Wojcieszak et al. 2018b). A higher dose of α-PVP (25 mg/kg), given orally also produced an increase of extracellular DA levels in the mouse striatum with a lower maximal effect (ca. 400%), but this observation may be also strongly attributed to a different route of administration and thus a strong impact of pharmacokinetics (Kaizaki et al. 2014). Both MC and 3-FMC differ from α-PVP also in their influence on the serotoninergic transmission, since their effects on extracellular 5-HT levels in the mouse striatum seem to have a shorter duration compared to α-PVP, which at 10 mg/kg produced a stable 5-HT increase during 180 min with no downward trend during the whole measurement period (Wojcieszak et al. 2018b). Taken together, these observations may suggest that methcathinone and its halogen-substituted analogs are capable of inducing extracellular 5-HT elevation in vivo.

## Conclusions

This study confirms that methcathinone and its halogenated derivative, 3-fluoromethcathinone increase spontaneous locomotor activity in mice. This effect is mediated via dopaminergic stimulation, as it was abolished by the blockade of D_1_-dopamine receptors with SCH 23390. Although methcathinone and its halogenated analogs are considered as selective DA and NE uptake inhibitors and releasers with little activity on 5-HT uptake, MC and 3-FMC produce significant increases of both extracellular DA and 5-HT levels in the mouse striatum, an effect that is probably mediated by the increased release of monoamines stored inside nerve terminals, presumably due to the functional DA—5-HT crosstalk. Finally, effects of both MC and 3-FMC observed in the assessment of spontaneous locomotor activity and extracellular DA and 5-HT levels in mice are similar to previously reported effects of methamphetamine, an observation that further supports their classification as methamphetamine-like cathinones.
